# Use of Integra for Reconstruction after Nevi Resection: A Systematic Review and Pooled Analysis of Reported Cases

**DOI:** 10.1155/2019/9483627

**Published:** 2019-10-09

**Authors:** Jude Opoku-Agyeman, Kayla Humenansky, Wellington Davis, Paul Glat

**Affiliations:** ^1^Department of Plastic Surgery, Philadelphia College of Osteopathic Medicine, Philadelphia, PA, USA; ^2^Division of Plastic Surgery, St. Christopher's Hospital for Children, Philadelphia, PA, USA

## Abstract

**Background:**

The use of Integra Dermal Reconstruction Template has emerged as an option for wound reconstruction, after resection of congenital nevi, especially giant congenital nevi. There have been many reports on Integra use in the literature for this purpose. This systematic review with pooled analysis examines the current literature regarding Integra use after resection of congenital nevi, including patient characteristics and reported outcomes.

**Methods:**

Systematic electronic searches were performed using PubMed, Ovid, Embase, and Cochrane library databases for studies reporting the use of Integra to reconstruct defects after nevi resection. Studies were analyzed if they met the inclusion criteria. Pooled descriptive statistics were performed.

**Results:**

Thirteen studies that met the inclusion criteria were included for analysis, yielding 31 procedures in 31 patients. Eleven of the thirteen studies were case reports representing 17 of the 31 patients. One study was retrospective, and the other study was a prospective study. The mean follow-up was 2.67 years (range, 0.2–13 years). The overall wound closure rate was 100%. The overall initial Integra take rate was 90.3% and the skin graft take rate was 100%. The rate of reported complications was 14.8%. The average age of patients was 7.36 years. The average size of the nevus was 6.29% TBSA (range, 0.005%–26%), and the time to definitive skin grafting was 3.28 weeks. Significant heterogeneity was found among the published studies.

**Conclusion:**

We conclude that the use of Integra appears to be a safe and viable option for defect reconstruction after the primary or secondary excision of congenital nevi of different sizes and on most parts of the body. Long-term follow-up studies and prospective cohort studies are required in order to fully estimate the incidence of complications. However, the rarity of this condition make these types of studies very difficult.

## 1. Introduction

Congenital melanocytic nevi (CMN) are benign proliferations of melanocytic cells in the epidermis or dermis. The estimated incidence of CMN in the general population is in the vicinity of 1 to 2 percent [1]. The incidence of giant hairy melanocytic melanoma, a subset of CMN, is even rarer. A major concern regarding patients with CMN is the possibility of developing melanoma. There is irrefutable evidence in the literature to support the increased risk of developing melanoma in individuals with CMN compared with the general population. A systematic review conducted by Krengel et al. in 2006 found the incidence of melanoma to be 0.7 percent in patients with CMN [2]. Another study in 2005 by Bett showed a 2.9 percent incidence of melanoma in patients with garment large CMN and 0.3 percent in patients with head and neck large CMN [3].

The risk of melanoma has been associated with the size of the nevus. Giant CMN, CMN greater than 20 cm in size, carry the greatest risk of melanoma transformation [2]. Patients with CMN are often referred to plastic surgeons for excision due to cosmetic concerns and the associated risk of malignant degeneration. The recommended treatment for these lesions is complete resection of the involved skin and tissue [4].

The surgical treatment of CMN can be very challenging depending on the size and location of the lesion. Resection of CMN, especially medium or giant CMN, can leave a sizeable defect that often requires major reconstructive surgery. Techniques available include local tissue rearrangement, full-thickness skin grafting (FTSG), and split-thickness skin grafting (STSG) as well as tissue expansion with subsequent defect closure in at least two-step procedures. Free tissue transfer can also be used as part of the reconstructive ladder.

The Integra bilayer wound matrix was developed in the early 1980s by Yannas et al. [5, 6]. Since its introduction, it has been used in a variety of wound reconstructions [7]. There have also been reports of Integra use in wound reconstruction after resection of CMN. Some of the reported advantages of Integra include less donor site morbidity compared to FTSG, STSG, and major flap reconstruction as well as decreased rate of wound contracture and hypertrophic scars. The purpose of this study was to review the current literature on the use of Integra for reconstruction after resection of CMN and to analyze patient characteristics and outcomes using pooled data analysis.

## 2. Methods

### 2.1. Search Methodology

Electronic searches were performed using the PubMed, Ovid, Embase, and Cochrane library databases for studies reporting on the application of Integra for congenital nevus reconstruction. The search keywords and terms include the following: “nevus,” “nevus surgery,” and “Integra.” All studies published up to February 2019 were thoroughly reviewed and the references of those articles were reviewed for additional relevant studies.

### 2.2. Selection Criteria

**Figure 1:**
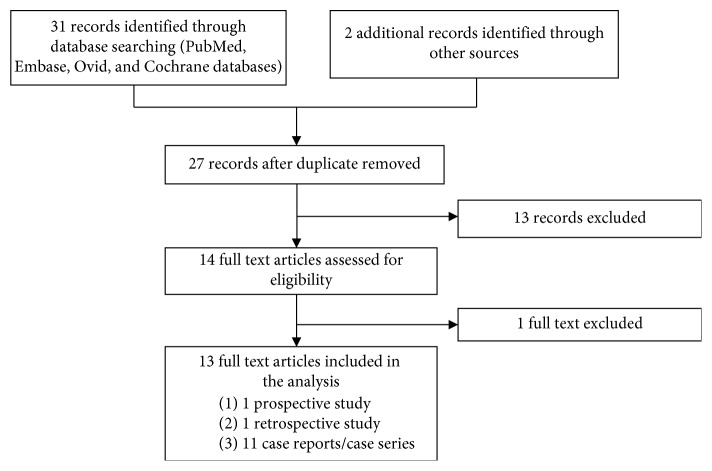
Flow chart of the literature search.

The inclusion and exclusion criteria were defined before the initiation of data collection. Studies that reported on the use of Integra for reconstruction of defects after resection of CMN were included. Retrospective, prospective, and case reports published in peer-reviewed journals were included.  Figure 1 outlines the selection process. The case reports must include at least the size of the lesion or a picture of the lesion, the type of skin coverage used, and the outcome of wound closure. If not, it was excluded. Studies were also excluded if they were not written in English, or were commentaries, reviews, or letters.

### 2.3. Data Collection and Analysis

The literature search, data extraction, and assessment of inclusion were conducted by two of the authors (JO and KH) with uncertainties resolved through discussion. The following data were collected: author, publication year, type of study, and number of patients. The primary data collected from the articles included the age of the patient at the time of resection and Integra application, gender, size of the lesion expressed as percentage total body surface area (%TBSA). For the cases where the size of the lesion was not reported in %TBSA, the patient's image was reviewed, and the size of the lesion was converted to %TBSA using the Lund and Brower chart. We also collected data on the location of the lesion and the initial Integra take rate, defined as incorporation of the Integra without the need for reapplication. The type of skin graft coverage used was collected along with the initial skin graft take rate, which was defined as graft integration without the need for regrafting. We also collected data on complications, if reported, the time of follow-up, and data on whether the wound achieved eventual closure without using other rescue modalities, such as local flaps, regrafting, or wound VAC therapy.

## 3. Results

Thirteen studies were included in the analysis.  Table 1 presents the characteristics of the studies that were included in the analysis. Almost all the reported cases were case reports, accounting for 54.8% of the patients [4, 9–16, 18, 19]. Of the remaining studies, one was retrospective [17] and one study was a prospective study [8]. The thirteen studies yielded 31 procedures in 31 patients. In terms of follow-up, 2 of the studies did not report any follow-up period [16, 17]. Most of the studies indicated whether or not there were any complications, but three of the studies did not mention the presence or absence of any complications [13, 14, 16]. Furthermore, 4 studies did not report the time from Integra placement to skin grafting [10, 13, 16, 17].

Table 2 represents the patient characteristics and reported outcomes. The average age was 6.97 ± 8.00 years, with 58.1% of patients being males and 41.9% being females. The trunk was the highest reconstructed area (51.9%), followed by the extremities (32%) and the face (16.1%). The average size of nevus resected was 6.29 ± 5.73%TBSA, with a range of 0.005–26% TBSA. The initial Integra take rate was 90.3%. The average time from Integra placement to skin grafting was 3.28 ± 0.83 weeks.

The initial Integra take rate was 90.3%, with an initial skin graft take rate of 100%. Skin grafting was achieved with mostly split-thickness skin graft in 83.9%, but one study utilized full-thickness skin graft, representing 6.5% of the grafts [17], and 2 studies used cultured epithelial autografts, representing 9.7% of the grafts [10, 16]. Complete wound closure and healing occurred in 100% of the patients. Complication rate was 14.8%. The mean follow-up was 2.56 years (range, 0.2–13 years).

## 4. Discussion

This literature review included 13 unique studies yielding 31 procedures involving the application of Integra after resection of congenital nevi. The data extracted in the review is best estimated from large prospective studies; however, these data are rare. The first reported case of Integra for this purpose was in 2003 by Kopp et al. [11, 12]. Since then, there have been a few reported cases in the English literature. Most of the reported cases are case reports with a single prospective study. There was significant heterogenicity in the reported cases. All the cases reported on the age of the patient, location of the lesion, initial Integra take, type of skin graft, initial skin graft take, and results of wound healing. The size of the lesions was reported using diameter measurement in some cases and %TBSA in others. Some cases did not mention the size of the lesion but provided a picture of the lesion. Not all studies reported on the time from Integra placement to skin grafting, follow-up time, and complication rate.

The average age of the resection and reconstruction was 7.36 years. Almost all the cases were performed on pediatric patients, except for one adult patient. This observation is in line with the recommended early resection of the lesions in childhood. Majority of the lesions in this review were located on the trunk, followed by the extremities, then head, and neck. This is in accordance with the reported distribution of congenital nevus on the body [20]. Based on %TBSA, lesions greater than 2% TBSA are classified as giant CMN [21]. The average size of the resected lesions in this study was 6.29% TBSA, with the largest reported lesion of 26% TBSA and the smallest lesion of about 0.005% TBSA. Based on this, one can infer that the average lesion reconstructed with Integra is a giant congenital nevus. It also demonstrates that Integra is feasible in the reconstruction of small defects created by excision of CMN as well.

Loss of Integra integration is a reported complication of the use of Integra. We defined the initial Integra take rate as any amount of Integra take that does not require reapplication of the whole or part of the Integra to achieve optimum closure. Our pooled analysis showed an Integra take rate of 90.3%. For studies that reported the time to skin grafting, the average time of Integra application to the application of skin graft was 3.28 weeks. Although most of the skin grafts were STSG (83.9%), FTSG and CEA were also applied successfully to achieve complete wound closure. Once the skin graft was applied, there was a 100% initial take rate. All the wound achieved complete healing with a mean follow-up of 2.56 years.

For the studies that reported any form of complication, the combined complication rate was 14.8%, including 1 infection, 1 Integra nonintegration, 1 skin regrafting, and 1 hypertrophic scar.

A systematic review conducted by Vourc'h-Jourdain et al. found out that the risk of developing melanoma from large CMN was about 1 in 20,00 to 1 in 50,000 and that the treatment of these lesions should not be generalized but rather individualized. This individual assessment should take into consideration the risk of malignant transformation as well as psychosocial and esthetic issues [22].

There are several limitations in this review and analysis. Only case reports, prospective studies, and retrospective studies were included in the analysis. There were no randomized controlled studies reported on this topic. Moreover, the quality of reporting in the included case reports was generally low. Some studies did not report on any complications. In spite of all these limitations, the combined data demonstrated the potential viability and safety of Integra in reconstruction of defects created after excision of CMN.

## Figures and Tables

**Table 1:** Characteristics of the included studies.

References	Type of study	Year of publication	Number of patients
Schiestl et al. [8]	Prospective	2009	12
Ramasamy and Jeffery [9]	Case report	2008	1
Ozerdem et al. [10]	Case report	2003	1
Kopp et al. [11]	Case report	2003	1
Kumbla et al. [12]	Case report	2015	2
Maguire et al. [13]	Case report	2017	1
Barcot et al. [14]	Case report	2017	1
Alrawi and Jeffery [15]	Case report	2009	2
Earle and Marshall [16]	Case report	2005	2
Grunwaldt et al. [17]	Retrospective	2013	2
Abai et al. [4]	Case report	2004	4
Marano et al. [18]	Case report	2015	1
Tønseth et al. [19]	Case report	2016	1

**Table 2:** Pooled data from all 13 included studies.

Patient	Age (yrs)	Gender	Site	Size (%TBSA)	Initial Integra take rate	Time to skin graft (weeks)	Type of skin coverage	Follow-up time (yrs)	Initial skin graft take rate	Complete wound healing	Complications
Schiestl et al. [8]	2.2	F	Face	2	Yes	3.1	STSG	4.08	Yes	Yes	None
	11.0	F	Face	3	Yes	3.1	STSG	4	Yes	Yes	None
	8.3	F	Trunk	2	Yes	2.7	STSG	3.6	Yes	Yes	None
	3.5	F	Trunk	10	No	4.3	STSG	0.08	Yes	Yes	Infection
	0.6	F	Trunk	2	No	2.7	STSG	2.92	Yes	Yes	No Integra take
	0.8	M	Trunk	12	Yes	2.1	STSG	2.35	Yes	Yes	None
	3.8	M	Arm	3	Yes	3	STSG	2	Yes	Yes	None
	2.5	M	Leg	3	No	2.7	STSG	1.5	Yes	Yes	None
	1.2	M	Face	1	Yes	3.1	STSG	1.35	Yes	Yes	None
	9.7	F	Trunk	4	Yes	2.9	STSG	1.15	Yes	Yes	None
	2.3	F	Arm	4	Yes	2.3	STSG	0.75	Yes	Yes	None
	1.5	F	Trunk	6	Yes	3	STSG	0.5	Yes	Yes	None

Tønseth et al. [19]	1.5	M	Trunk	16	Yes	3	STSG	0.2	Yes	Yes	Regrafting

Ramasamy and Jeffery [9]	17.0	M	Trunk	10	Yes	6	STSG	0.8	Yes	Yes	None

Ozerdem et al. [10]	6.0	M	Trunk	6	Yes	—	CEA	0.08	Yes	Yes	None

Kopp et al. [11]	44.0	F	Trunk	12	Yes	2.9	STSG	2.5	Yes	Yes	None

Kumbla et al. [12]	5.5	F	Hands/arm	2	Yes	3	STSG	1	Yes	Yes	None
	3.7	F	Hands/arm	2	Yes	3	STSG	1	Yes	Yes	None

Maguire et al. [13]	0.4	F	Trunk	26	Yes	—	STSG	5	Yes	Yes	—

Barcot et al. [14]	9.0	F	Leg/foot	5	Yes	3	STSG	2.7	Yes	Yes	—

Alrawi and Jeffery [15]	13.0	M	Trunk	16	Yes	4	STSG	13	Yes	Yes	None
	12.0	M	Thigh/leg	12	Yes	4	STSG	13	Yes	Yes	None

Earle and Marshall [16]	7.0	F	Thigh	3	Yes	—	CEA	—	Yes	Yes	—
	8.0	M	Trunk	12	Yes	—	CEA	—	Yes	Yes	—

Grunwaltd et al. [17]	11.0	M	Nose	0.005	Yes	—	FTSG	—	Yes	Yes	None
	7.0	F	Nose	0.005	Yes	—	FTSG	—	Yes	Yes	None

Abai et al. [4]	2.8	F	Leg	4	Yes	4	STSG	0.5	Yes	Yes	None
	13.0	M	Leg	4	Yes	4	STSG	1	Yes	Yes	None
	10.0	F	Trunk	4	Yes	4	STSG	2	Yes	Yes	None
	8.0	F	Trunk	6	Yes	4	STSG	1.1	Yes	Yes	None

Marano et al. [18]	2.0	M	Trunk	3	Yes	2	STSG	1	Yes	Yes	Hypertrophic scar

Totals	Mean: 7.36 yrs SD: 7.99 yrs	M: 77% F:33%	Trunk: 51.6% Extremities: 32.3% Face: 16.1%	Mean: 6.29% TBSA SD: 5.73% TBSA	90.30%	Mean: 3.28 wks SD: 0.83 wks	STSG: 83.9% FTSG: 6.5% CEA: 9.7%	Mean: 2.56 yrs SD: 3.22 yrs	100%	100%	14.8%

## Supplementary Materials

Supplementary Materials Excluded studies and literature search strategy.

## References

[1] Kroon S., Clemmensen O. J., Hastrup N. (1987). Incidence of congenital melanocytic nevi in newborn babies in Denmark. *Journal of the American Academy of Dermatology*.

[2] Krengel S., Hauschild A., Schafer T. (2006). Melanoma risk in congenital melanocytic naevi: a systematic review. *British Journal of Dermatology*.

[3] Bett B. J. (2005). Large or multiple congenital melanocytic nevi: occurrence of cutaneous melanoma in 1008 persons. *Journal of the American Academy of Dermatology*.

[4] Abadi B., Thayer D., Glat P. M. (2004). The use of a dermal regeneration template (integra) for acute resurfacing and reconstruction of defects created by excision of giant hairy nevi. *Plastic and Reconstructive Surgery*.

[5] Yannas I., Burke J., Orgill D., Skrabut E. (1982). Wound tissue can utilize a polymeric template to synthesize a functional extension of skin. *Science*.

[6] Yannas I. V., Lee E., Orgill D. (1989). Synthesis and characterization of a model extracellular matrix that induces partial regeneration of adult mammalian skin. *Proceedings of the National Academy of Sciences*.

[7] Besner G. E., Klamar J. E. (1998). Integra artificial skin∗ as a useful adjunct in the treatment of purpura fulminans. *Journal of Burn Care & Rehabilitation*.

[8] Schiestl C., Stiefel D., Meuli M. (2010). Giant naevus, giant excision, eleg(i)ant closure? Reconstructive surgery with integra artificial skin® to treat giant congenital melanocytic naevi in childrenficial skin to treat giant congenital melanocytic naevi in children. *Journal of Plastic, Reconstructive & Aesthetic Surgery*.

[9] Ramasamy A., Jeffery S. L. A. (2008). The use of a dermal regeneration template following excision of a giant melanocytic nevus in a potential army recruit. *Military Medicine*.

[10] Ozerdem O. R., Wolf S. A., Marshall D. (2003). Use of skin substitute in pediatric patients. *The Journal of Craniofacial Surgery*.

[11] Kopp J., Magnus Noah E., Rübben A., Merk H. F., Pallua N. (2003). Radical resection of giant congenital melanocytic nevus and reconstruction with meek-Graft covered integra dermal template. *Dermatologic Surgery*.

[12] Kumbla P. A., Yuen J. C., Tait M. A. (2015). Applying a dermal regenerative template in management of congenital melanocytic nevi of the hand. *Plastic and Reconstructive Surgery—Global Open*.

[13] Maguire C. R., Livingston R., Phillips G. E., Kimble R. M. (2017). Giant congenital melanocytic nevi and malignant transformation: a case for early radical intervention. *Pediatric Surgery International*.

[14] Barcot Z., Inga D. B., Zupancic B., Bacalja V. (2017). Treating giant congenital nevus with integra dermal regeneration template in a 9-year-old girl. *The International Journal of Lower Extremity Wounds*.

[15] Alrawi M. F., Jeffery S. L. (2009). The surgical challenge of giant circumferential congenital naevi of the extremities: a 13-year follow-up of two cases. *European Journal of Plastic Surgery*.

[16] Earle S. A., Marshall D. M. (2005). Management of giant congenital nevi with artificial skin substitutes in children. *The Journal of Craniofacial Surgery*.

[17] Grunwaldt L. J., Adetayo O. A., MacIsaac Z. M., Losee J. E., Kumar A. R. (2014). Successful reconstruction of complex pediatric nasal lesions. *Plastic and Reconstructive Surgery Global Open*.

[18] Marano A. A., Feintisch A. M., Datiashvili R. (2015). Giant congenital melanocytic nevus of the buttock. *Eplasty*.

[19] Tønseth K. A., Filip C., Hermann R., Vindenes H., Høgevold H. (2015). Extraordinary large giant congenital melanocytic nevus treated with integra dermal regeneration template. *Plastic and Reconstructive Surgery—Global Open*.

[20] Egan C. L., Oliveria S. A., Elenitsas R., Hanson J., Halpern A. C. (1998). Cutaneous melanoma risk and phenotypic changes in large congenital nevi: a follow-up study of 46 patients. *Journal of the American Academy of Dermatology*.

[21] Shah J., Feintisch A. M., Granick M. S. (2016). Congenital melanocytic nevi. *Eplasty*.

[22] Vourc’h-Jourdain M., Martin L., Barbarot S. (2013). Large congenital melanocytic nevi: therapeutic management and melanoma risk. *Journal of the American Academy of Dermatology*.

