# Supplementary material for: Use of Integra for Reconstruction after Nevi Resection: A Systematic Review and Pooled Analysis of Reported Cases
Source: Surg Res Pract. 2019 Oct 9;2019:9483627. doi: 10.1155/2019/9483627 (PMC6803724; doi:10.1155/2019/9483627)
Supplement: Supplementary Materials — Excluded studies and literature search strategy. [file 9483627.f1.pdf]

Supplementary material

Excluded studies and reasons they were excluded

1. Grufman V, Fritsche E, Hug U. Treatment of a frontotemporal congenital melanocytic nevus combining Integra® with split-thickness skin grafting. Handchir Mikrochir Plast Chir. 2018 Dec;50(6):433-434. doi: 10.1055/s-0044-101709. Epub 2019 Jan 8.

Reason for exclusion: Paper in German

2. Bellier-Waast F1, Perrot P, Duteille F, Stalder JF, Barbarot S, Pannier M. Surgical treatment for giant congenital nevi: what are the psychosocial consequences for the child and family? Ann Chir Plast Esthet. 2008 Oct;53(5):408-14. doi: 10.1016/j.anplas.2007.10.002. Epub 2008 Mar 20.

Reason for exclusion: Article is in French

3. Chatrian L. Margaux, a little girl with a giant nevus. Soins Pediatr Pueric. 2005 Dec;(227):18-9.

Reason for exclusion: Article is in French

4. Molnar JA1, DeFranzo AJ, Hadaegh A, Morykwas MJ, Shen P, Argenta LC. Acceleration of Integra incorporation in complex tissue defects with subatmospheric pressure. Plast Reconstr Surg. 2004 Apr 15;113(5):1339-46.

Reason for exclusion: Did not involve nevus resection

5. Martínez L1, Ros Z, López-Gutiérrez JC, Díaz M, Quezada B, Perdiguero M, Hernández F, Rivas S, Tovar JA. Integra Artificial dermis in pediatric reconstructive surgery. Cir Pediatr. 2002 Jul;15(3):97-100.

Reason for exclusion: Article in Spanish

6. Chan ES1, Lam PK, Liew CT, Lau HC, Yen RS, King WW. A new technique to resurface wounds with composite biocompatible epidermal graft and artificial skin. J Trauma. 2001 Feb;50(2):358-62.

Reason for exclusion: The article does not involve the resection of a nevus.

7. Arneja, Jugpal S. M.D.; Gosain, Arun K. M.D Giant Congenital Melanocytic Nevi. Plastic and Reconstructive Surgery: August 2007 - Volume 120 - Issue 2 - p 26e-40e

Reason for exclusion: Review study

8. Rajiv Sood, Jerone Balledux, Dimitri J. Koumanis, Haaris S. Mir, Swetanshu Chaudhari, David Roggy, Madeline Zieger, Adam Cohen, John J. Coleman, Coverage of Large Pediatric Wounds With Cultured Epithelial Autografts in Congenital Nevi and Burns: Results and Technique, *Journal of Burn Care & Research*, Volume 30, Issue 4, July-August 2009, Pages 576–586,

Reason for exclusion: Integra was not used.

9. Amir Ismail, Kristiina Jarvi Alessandra, C.E. Canal. Successful resurfacing of scars from previous deliberate self-harm using Integra dermal matrix substitute. Journal of Plastic, Reconstructive & Aesthetic Surgery Volume 61, Issue 7, July 2008, Pages 839-841

Reason for exclusion: Does not involve excision of nevus

10. A. Chokoeva, M. Fioranelli, M.G. Roccia, T. Lotti, U. Wollina , G. Tcherne. Giant congenital melanocytic nevus in a Bulgarian newborn. Journal of biological regulators and homeostatic Vol. 30, no. 2 (S2), 0-0 (2016)

Reason for exclusion: Integra was not used in the reconstruction

11. Sidonia Popescu, N. Ghetu, Oana Grosu, Mihaela Nastasa, D. Pieptu. IntegraTM - a therapeutic alternative in reconstructive surgery. Our first experience. Chirurgia (Bucharest, Romania: 1990) · March 2007

Reason for exclusion: No nevus was resected

12. Francky Teddy Endomba, Charlie Romain Mbega, Joel Noutakdie Tochie & Saint-Just N. Petnga. Giant congenital melanocytic nevus in a Cameroonian child: a case report. Journal of Medical Case Reports volume 12, Article number: 175 (2018) |

Reason: The nevus was not reconstructed with Integra.

13. Su, Jeannie J. MAS; Chang, Daniel K. MD; Mailey, Brian MD; Gosman, Amanda MD. Treatment of a Giant Congenital Melanocytic Nevus in the Adult. Review of the Current Management of Giant Congenital Melanocytic Nevus Annals of Plastic Surgery: May 2015 - Volume 74 - Issue - p S57–S61.doi: 10.1097/SAP.0000000000000433

Reason for exclusion: nevus was reconstructed with a combination of flaps and integra.

Search strategy used in PubMed

("naevus"[All Fields] OR "nevus, pigmented"[MeSH Terms] OR ("nevus"[All Fields] AND "pigmented"[All Fields]) OR "pigmented nevus"[All Fields] OR "nevus"[All Fields] OR "nevus"[MeSH Terms]) AND ("naevus"[All Fields] OR "nevus, pigmented"[MeSH Terms] OR ("nevus"[All Fields] AND "pigmented"[All Fields]) OR "pigmented nevus"[All Fields] OR "nevus"[All Fields] OR "nevus"[MeSH Terms]) AND ("surgery"[Subheading] OR "surgery"[All Fields] OR "surgical procedures, operative"[MeSH Terms] OR ("surgical"[All Fields] AND "procedures"[All Fields] AND "operative"[All Fields]) OR "operative surgical procedures"[All Fields] OR "surgery"[All Fields] OR "general surgery"[MeSH Terms] OR ("general"[All Fields] AND "surgery"[All Fields]) OR "general surgery"[All Fields]) AND integra[All Fields
